# Staff experiences of training and delivery of remote home monitoring services for patients diagnosed with COVID-19 in England: A mixed-methods study

**DOI:** 10.1177/13558196231172586

**Published:** 2023-06-27

**Authors:** Manbinder Sidhu, Holly Walton, Nadia Crellin, Jo Ellins, Lauren Herlitz, Ian Litchfield, Efthalia Massou, Sonila M Tomini, Cecilia Vindrola-Padros, Naomi J Fulop

**Affiliations:** 1 Associate Professor, Health Services Management Centre, School of Social Policy, 1724 University of Birmingham, UK; 2 Research Fellow, Department of Applied Health Research, 4919 University College London, UK; 3 Fellow, 4853 Nuffield Trust, London, UK; 4 Senior Fellow, Health Services Management Centre, School of Social Policy, 1724 University of Birmingham, UK; 5 Research Fellow, Population, Policy and Practice, Great Ormond Street Institute of Child Health, University College London, UK; 6 Senior Research Fellow, Institute of Applied Health Research, 150183 College of Medical and Dental Sciences, University of Birmingham, UK; 7 Research Associate, Department of Public Health and Primary Care, 2152 University of Cambridge, UK; 8 Assistant Professor, Global Business School for Health, 4919 University College London, UK; 9 Professorial Research Fellow, Department of Targeted Intervention, 4919 University College London, UK; 10 Professor of Health Care Organisation and Management, Department of Applied Health Research, 4919 University College London, UK

**Keywords:** COVID-19, remote monitoring, staff experience

## Abstract

**Objectives:**

Remote home monitoring services for patients at risk of rapid deterioration introduced during the COVID-19 pandemic had important implications for the health workforce. This study explored the nature of ‘work’ that health care staff in England undertook to manage patients with COVID-19 remotely, how they were supported to deliver these new services, and the factors that influenced delivery of COVID-19 remote home monitoring services for staff.

**Methods:**

We conducted a rapid mixed-methods evaluation of COVID-19 remote home monitoring services during November 2020 to July 2021 using a cross-sectional survey of a purposive sample of staff involved in delivering the service (clinical leads, frontline delivery staff and those involved in data collection and management) from 28 sites across England. We also conducted interviews with 58 staff in a subsample of 17 sites. Data collection and analysis were carried out in parallel. We used thematic analysis to analyse qualitative data while quantitative survey data were analysed using descriptive statistics.

**Results:**

A total of 292 staff responded to the surveys (39% response rate). We found that prior experience of remote monitoring had some, albeit limited benefit for delivering similar services for patients diagnosed with COVID-19. Staff received a range of locally specific training and clinical oversight along with bespoke materials and resources. Staff reported feeling uncertain about using their own judgement and being reliant on seeking clinical oversight. The experience of transitioning from face-to-face to remote service delivery led some frontline delivery staff to reconsider their professional role, as well as their beliefs around their own capabilities. There was a general perception of staff being able to adapt, acquire new skills and knowledge and they demonstrated a commitment to continuity of care for patients, although there were reports of struggling with the increased accountability and responsibility attached to their adapted roles at times.

**Conclusions:**

Remote home monitoring models can play an important role in managing a large number of patients for COVID-19 and possibly a range of other conditions. Successful delivery of such service models depends on staff competency and the nature of training received to facilitate effective care and patient engagement.

## Introduction

Remote home monitoring services have received increasing attention by policy makers as a means to address rising demand for health care because of population ageing and higher burden of chronic health conditions as well as to empower patients to take greater responsibility for their own care.^1,2^ Remote home monitoring offers potential advantages to patients, such as increased engagement or reduced need for travel to appointments, and the wider health care system, enabling a more agile response, for example, during pandemics, and, possibly, cost savings.^3^ At the same time, remote home monitoring may be clinically risky, less acceptable to patients, and it may bring significant technical, logistical and regulatory challenges.^4^ Our patient experience study of COVID-19 remote home monitoring services highlighted that patients generally found the service reassuring but that there were some barriers to engagement.^5^ Also, the use of technology as part of remote monitoring service models can be difficult for some population groups, such as those considered to be vulnerable.^6^

There is a dearth of evidence of how remote home monitoring service models are experienced by health care staff and there remains a need for best practice guidelines to support staff.^6^ Evidence has highlighted impacts on job satisfaction (positive and negative);^7,8^ yet, there are concerns over ‘call-centre medicine’,^9^ and lack of a supportive infrastructure available to staff, such as appropriate training, clinical guidance and technological support,^10^ as well as the exclusion of some service user groups if alternatives to remote care are not provided.^11^

Delivering remote home monitoring services requires staff to complete a range of clinical and non-clinical activities, from determining patient eligibility, to providing monitoring equipment and instructions, symptom monitoring, and discharge.^12^ A major concern has been around the ability to make accurate assessments of patients’ physical well-being at distance. Health professionals thus engage in a form of ‘risk work’ when making autonomous clinical decisions remotely.^13,14^ Risk work is defined by three key features: (1) interpreting risk knowledge (i.e., based on evidence and/or past experience which informs medical decision making); (2) intervening to reduce risk (i.e., reworking risk knowledge into action with both intended and unintended consequences); and (3) handling social relations and interactions (i.e., conflicts of role and tensions in the health professional-service user relationship). We used this conceptual understanding to assess staff experience of delivering COVID-19 remote home monitoring services in England. Specifically, we sought to understand the nature of ‘work’ staff undertake to manage patients with COVID-19, how staff are supported to deliver remote home monitoring services, and the barriers and facilitators influencing delivery of COVID-19 remote monitoring services (Box 1).

**Box 1. COVID-19 remote home monitoring services**

COVID-19 remote home monitoring services seek to remotely monitor patients considered at high risk of deterioration at home to: reduce infection transmission, avoid unnecessary hospital admission, and enable cases of deterioration to be escalated at an earlier stage to avoid invasive ventilation and admission to intensive care. Within these models, patients are triaged, provided with equipment and information, are remotely monitored regularly by a team of staff and escalated as necessary, and finally discharged from the service.^12^ Related services are implemented in several countries; ^12^ they mostly use a pulse oximeter to measure blood oxygen saturation levels, with readings submitted via the telephone (analogue services) and/or the use of technology-enabled methods (digital applications, web links, or automated texts or calls).

In England, several primary and secondary care services began setting up remote home monitoring services to monitor COVID-positive patients during the first wave of the COVID-19 pandemic in spring 2020 supported by national government (NHS England) and local health care organisations and networks. The service was rolled out nationally by the start of a second wave in November 2020 using two types of approaches: COVID Oximetry@home (CO@H, in November 2020) and COVID virtual wards (CVW, in January 2021). In COVID Oximetry@home models, patients are referred via community health settings (such as GP surgeries, COVID-19 hot hubs or emergency departments). In virtual ward models, patients are referred upon early discharge from hospital.^15^

## Methods

We conducted a rapid mixed-methods evaluation of COVID-19 remote home monitoring services during November 2020 to July 2021, using a cross-sectional survey and qualitative interviews with clinical and non-clinical staff delivering COVID-19 remote home monitoring services (see Online Supplement S1 for details).

### Patient and public involvement

Study design was informed by a Public and Patient Involvement (PPI) advisory group including service users, patient representatives and public members to discuss the study and identify research questions and methods of recruitment to ensure inclusivity (four 90-minute meetings from November 2020 to July 2021). The advisory group reviewed participant-facing documents, including consent forms, topic guides, survey, and information sheets, with feedback incorporated into the study documents prior to data collection. Online meetings were held throughout data analysis and write up to share learning and cross-check author interpretations.

### Sample

We recruited staff from 28 sites operating remote home monitoring services for COVID-19 patients across England. We purposively sampled sites for maximum variation using a range of criteria such as geographic location, setting (primary care, secondary care or both), type of model (COVID Oximetry@home or COVID virtual ward; see Box 1), mechanism for patient observation reporting (technology-enabled, paper-based or both) and stage of implementation (whether their model was implemented in wave 1 of the pandemic in England (March to August 2020), wave 2 (October 2020 to February 2021), or both). Sites represented all English regions (Online Supplement S2). Twenty-eight sites were survey sites, and seventeen of these were case study sites (interview and survey data).

Surveys and interviews were conducted with a purposive sample of clinical leads (senior clinical and/or doctor equivalent roles including senior nurses and consultants), frontline delivery staff (nursing and allied health professionals) and those involved in data collection and management (‘data leads’ such as managers, non-clinical administrative staff and volunteers). For the survey, we aimed to recruit as many staff as possible and for interviews we aimed to speak with up to four members of staff from each service.

### Measures

We iteratively developed the survey and semi-structured topic guide steered by relevant literature (Online Supplement S1) and our research questions. We piloted the topic guide and survey with senior nurses delivering COVID-19 remote home monitoring and members from our PPI group (*n* = 3). Staff interviews and surveys focused on staff experiences of implementing and delivering the service. Each site recorded the number of surveys distributed to staff to determine response rates.

### Procedure

For the interviews, participants were approached by study coordinators (local clinical leads or managers of remote home monitoring services) from each site and asked if they were interested in taking part. Those expressing interest were contacted by a researcher who sent them an information sheet and consent form which participants were asked to return ahead of the interview. Interviews were conducted over Microsoft Teams or telephone. They lasted between 12 and 130 minutes. Interviews were audio recorded (subject to consent), transcribed verbatim by a professional transcription service, anonymised and kept in compliance with the General Data Protection Regulation (GDPR) 2018 and Data Protection Act 2018.

For the survey, study coordinators from each site distributed electronic surveys to staff involved in the service via email, followed by two reminders to facilitate completion. Members of staff completing the survey across sites remained anonymous to the study team.

### Analysis

For the interviews, data collection and analysis were carried out in parallel and facilitated through the use of Rapid Assessment Procedure (RAP) sheets.^16^ RAP sheets (collating the main points from interviews in real-time using a structured template) were developed per site to facilitate cross-case comparison. The categories used in the RAP sheets were based on the questions included in the interview topic guide, maintaining flexibility to add categories as the study was ongoing. RAP sheets were imported into qualitative analysis software (NVivo12) and an inductive thematic analysis was carried out. In addition, a sub-set of transcripts (*N* = 17, purposively selected to capture a range of experiences by type of model) were coded independently by two researchers (MS and HW) to validate interpretations from the RAP sheet analysis and until researchers were unable to identify any further themes.^17^ We then undertook a second cycle of analysis that was theoretically guided using the three concepts of ‘risk’, ‘risk-based knowledge’ and ‘risk work’ outlined by Gale et al.^13,14^ This was led by MS in discussion with co-authors, applying the concepts as a lens to identify and interpret instances of staff engaging with varying forms of knowledge and evidence upon which clinical decisions were made; how such decisions were communicated to other staff and patients; and how confident staff were in acting on clinical decisions based on such knowledge in a remote setting.

The survey data were analysed using SPSS statistical software (version 25). Descriptive statistics were used to explore staff survey responses. Where data were missing for specific questions, cases were excluded from the analysis and the denominator reported. Open text responses relating to staff experiences of delivering the service were coded thematically and inductively.

Qualitative interview data and quantitative survey data were triangulated to address the research questions.^18^ Within this study, interview and survey analysis were initially conducted separately before using data triangulation to compare the consistency of findings relating to the three research questions using both qualitative (interviews and survey open text) and quantitative (survey) methods.^19^

### Ethical approval

Ethical approval was granted by the University of Birmingham Humanities and Social Sciences Ethics Committee (ERN_13-1085AP39). The study was categorised as service evaluation by the University College London/University College London Hospital Joint Research Office (January 2021).

## Results

We received 292 surveys (39% response rate) from NHS staff (clinical leads or service managers: *n* = 70; delivery staff: *n* = 222; Online Supplement S3) across 28 sites delivering COVID-19 remote home monitoring services (CO@H: *n* = 13; CVW: *n* = 4; both: *n* = 11; Online Supplement S2). We completed interviews with 58 staff, including clinical leads (*n* = 23), delivery staff (*n* = 23), and data leads (*n* = 7) from across 17 sites (Online Supplement S4). The study team were unable to determine if those interviewed also completed the staff survey to preserve anonymity. We present interview findings here and survey results in Online Supplement S5, S6 and S7. We detail training and support received by staff in Online Supplement S8.

### The nature of ‘work and risk work’ staff undertake to manage patients with COVID-19 remotely

Staff were involved in remote monitoring services delivered in one of two ways: technology-enabled with analogue services (21/28 sites), and analogue only services (7/28 sites). Technology-enabled with analogue services included staff working with an app, making telephone calls with patients, and overseeing automated text messages to remind patients to provide readings. Analogue only services included staff making telephone calls with patients, with some services offering face-to-face visits.

Daily, staff would assess the nature of referrals made to the service, monitor data uploaded by patients about oxygen saturation, temperature, or breathlessness (as a minimum), and, if necessary, determine whether data warranted escalation in patient care (telephone call to the patient, sending paramedics to the patient, directing patients to seek emergency acute secondary care).

Delivery staff noted the absence of recording the wider socio-cultural impact the COVID-19 pandemic was having on patient lives as part of daily monitoring procedures. As a result, delivery staff used their own initiative to engage in conversations to understand the effects that the wider context of COVID-19 was having on patient lives, but such conversations were understood to be beyond the scope of daily monitoring and their role.

[W]e do, kind of, go outside the scope of nursing. But that’s, kind of, normal for any nursing role really. People will tell you different things. (Site L.2 Delivery staff)

### Support and training provided to staff to interpret and rework risk knowledge

We found that delivery staff provided a multi-faceted response to address challenges with monitoring, treating, and escalating care for patients. Many staff struggled with learning new skills and reworking information as part of triage and escalation processes despite showing high levels of confidence when monitoring patients as part of their daily activities. The greatest challenge for delivery staff was translating reworked information into confident clinical decisions when treating patients.

Additionally, interview findings showed that monitoring tasks were often completed using a range of platforms, many of which could not be integrated into existing working practices. This meant that greater time had to be taken by staff to upload patient data onto multiple databases, which was resource intensive, cumbersome, and increased the risk for potential mistakes. Thus, much ‘invisible work’ went into translating monitoring data and ‘re-embedding’ the information in a useable format.

Delivery staff, particularly nurses, raised concerns that most training led by senior clinicians took the form of shadowing or receiving brief overviews on each part of the service. As a result, initially many nurses reported a lack of confidence, especially when communicating risks associated with COVID-19, and remained largely dependent on senior clinicians. This dependency subsided as their experience grew. During this period, nurses welcomed that senior clinicians were readily accessible.

[…] you don’t need to know everything. You’re not a doctor. You just need to get all the information and you can always get a doctor to give them a call. […]. So, they’re fit for discharge. 99 times out of a hundred, they’re okay to be at home. So, don’t panic. Just, yes, reassure them and if you’re unsure, just get a doctor to give them a call. (Site L.1 Delivery staff)

### Factors influencing the reworking of risk knowledge and impact on social interactions

We identified a number of subthemes that demonstrate how service leads and delivery staff addressed the challenges associated with using risk knowledge to make clinical decisions and communicating risks in the context of a COVID-19 specific intervention. These were: multidisciplinary team dynamics; influences on staff workload; staff knowledge and confidence; and staff experience of patient engagement or disengagement. We discuss each in turn.

#### Multidisciplinary team dynamics

Where sites adopted a multidisciplinary team model of working, there were at times tensions between professional groups, especially where there was historically a lack of coworking. This remained problematic until services were more established and roles became more distinguished (e.g., GPs understanding that remote home monitoring services were not replacing their duty to continue doing home visits during the pandemic).

And I think it’s occasionally we’ve had GP practices that have referred patients for the virtual ward, for the COVID Oximetry @home. That really actually what they needed was a home visit. And we’ve bounced a couple of those back and we always do it by phone and we’ve hit some clashes with that. (Site I.4 Delivery staff)

The specific skills required to deliver care as part of remote monitoring models and establish rapport and relationships influenced the type of health professional that was considered appropriate by service leads to deliver the service. Staff with the necessary communication skills to undertake remote monitoring consultations via telephone, such as active listening, and picking up cues regarding a patient’s current condition without physical examination were prioritised by service leads for redeployment or recruitment. Key to handling social relations and conflicts in the health professional-service user relationship was the quality of communication. Our analysis of interview data found that the style and content of communication that staff adopted had important consequences for health professionals’ ability to grasp the complex dynamics of risk factors facing a particular patient with their illness. Delivery team members wanted flexibility, both in style and duration, regarding to how each call was made. Scripts and standard operating procedures were widely considered to be useful, but staff wanted to be able to provide patients with emotional support as well. This meant, at times, longer telephone calls to establish stronger social relations.

You spend a lot of your time actually reassuring patients that they’re doing all right. They’re frightened. They’re very frightened. And they always know somebody that’s been in hospital and has had a really bad experience. (Site I.4 Delivery staff)

#### Influences on staff workload

While most clinical leads or service managers felt there were enough staff and sufficient capacity to deliver the service as intended, delivery staff reported having to balance their role within the service with other roles. Service leads noted that delivery staff considered to be at high risk of infection, such as pregnant clinicians, were protected but were available to deliver the service when scale-up was needed, as these staff were often working solely on the remote home monitoring service. We found that staff across sites differentiated between working as part of a remote monitoring model and providing care on the frontline. The latter was centred on face-to-face interaction with patients while the former focused on establishing therapeutic relationships at a distance. Staff noted that many of the skills associated with face-to-face rapport building with patients were not transferable when establishing therapeutic relationships at a distance.

We identified notable differences in workloads between nurses that were redeployed meaning they were assigned a new role on a remote monitoring service, compared to nurses who were asked to take on additional work on top of their current roles. Clinical leads and delivery staff expressed concern that redeployed staff would not be available during future surges in patient cases.

So, the majority of our nursing team would be you know working all day in practice and then doing a couple of hours for us on an evening or popping in on a weekend. (Site I.3 Data staff)

We delivered on existing staffing. So it was mainly done via bank [an entity managed by a hospital, or through a third party organisation who contracts with health care staff to take on shifts at hospital or general practice]. We did have bank admin and bank drivers for deliveries and collections. [..] But it was done on existing staffing. So the staff that we already had stepped up, took on extra shifts, it was all done on bank and goodwill. (Site F.4 Delivery staff)

Understanding the impact on staff workload is important as the process of interpreting and intervening to make risk-based decisions is a collective practice between health professionals. Risk work can involve assessing risk, deciding and being accountable for said decisions as part of a team. For delivery staff, this process can be problematic when working across different clinical settings or being unfamiliar with the nuances of working as part of a remote monitoring model. Interpreting risk is fundamentally a social process and any tensions between health professionals because of workload demands could lead to tensions when communicating with patients.

#### Staff knowledge and confidence

Staff reported they would have been better prepared to deliver remote monitoring services if training to develop new knowledge, skills and confidence was delivered earlier as opposed to on-the-job learning. This was mainly because training was not always embedded in a timely way during service implementation. Some nursing staff felt unsupported and distressed at having to work on a remote home monitoring service. At one site staff initially ‘hated [the service] with a passion’ *(Site K.2, Delivery staff),* finding the responsibility of assessing patient risk too stressful and some interview participants stated that they considered leaving their roles unless improvements were made to their training. Thus, there was significant tension when services attempted to implement strategies to minimise risk and maximise safety in the face of underdeveloped training packages.

Meanwhile, some nursing staff felt sometimes to be ‘at risk’ of potentially making mistakes in relation to monitoring and decisions about potential escalation in the rapid scale-up of services as they had to rely on their previous experience of working with standard operating procedures. Delivery staff faced a balancing act between the negotiation of health risks as understood within the context of previous clinical experience and evolving evidence of how to treat COVID-19 patients as part of a remote monitoring model. Remote processes were perceived to be difficult to develop and implement as part of a ‘new’ remote workplace culture with risk assessment, interpretation, and potential escalation of patient care at its centre. However, there were some exceptions.

Working in A&E [Accident and Emergency] I can see some really acutely unwell patients and we use kind of an A to E approach [Airway, Breathing, Circulation, Disability and Exposure (ABCDE) approach to perform a systematic assessment of a critically unwell patient] where it’s kind of a system that we look at looking at airway, breathing, circulation etc. And then say when I’m speaking to a person on the phone, what we are concerned about is the breathing and things, so you’re kind of able to transfer that knowledge. (Site K.3 Delivery staff)

Caring for patients facing uncertain health outcomes is a key component of risk work. For delivery staff, such care came in the form of supporting people to make choices in the face of receiving a risk-based clinical assessment. In addition, we found that patients were also tasked with being responsible for adhering to treatment by delivery staff when managing their conditions and escalating care in the event of exacerbations. Delivery staff reported that they found it difficult to reconcile care with other aspects of risk work. This influenced the nature of trustworthy relationships built between professionals and patients.

#### Staff experience of patient engagement or disengagement

All interviewed staff reported that the delivery of remote home monitoring services was facilitated by patients engaging with the service and having confidence as well as sufficient digital literacy to submit their measurements and readings. Other reported facilitators included patient experience of managing long-term conditions. For example, those living with respiratory conditions such as chronic obstructive pulmonary disease (COPD) appeared to be more comfortable in dealing with episodes of breathlessness; these patients had a greater preference to receive information from nurses for managing COVID. At the same time, staff identified patient groups that they believed required more support and they found it more difficult to engage with them; these included older patients, those with health difficulties, and those with low digital literacy, as well as other groups likely to disengage with services (e.g., younger patients, those who had returned to work, or those with few acute symptoms associated with COVID-19). In response, staff across sites developed bespoke information sheets to support these patient groups as well as working around patients’ work commitments. Staff noted that in many cases, asking next of kin or family members to provide readings helped maintain engagement, which also facilitated effective delivery of the service. In addition, delivery staff reported that some patients preferred to provide oximetry readings with the support of a member of the remote home monitoring team over the telephone.

Reported barriers to delivering remote home monitoring services included: having to chase patients who did not submit readings, patients not answering the phone, delays in the provision of oximeters or other equipment, some patients struggling to use oximeters (e.g., unable to insert batteries, difficulties turning oximeter on, miscommunication of readings if a pulse reading was also required), patients completing compulsory isolation periods, asking family members to translate and complete tasks or readings on a patient’s behalf, some reluctance from care workers to engage in uploading readings due to an increased workload when caring for COVID-19 positive patients, some over-involvement of family members or advocates without consulting the patient first, and patients unwilling to answer calls from staff using withheld numbers.

But we do let people know, you know, we are going to be a withheld number and they- we do let them know the frequency of the calls, as well. So, when to expect us to call. But yes, some people won’t answer. So, that’s - which obviously, then, you know, limits what we can actually do for them. (Site L.2 Delivery staff)

Staff also felt that patients receiving too little written or oral information was a barrier to delivery as they then needed to implement additional processes to provide patients with more information about the service and how it was designed to help patients.

So basically we just give them more information about the service that we’re providing. We just explain that we usually call people on certain days, so like a first day, two, five, seven, ten, twelve and fourteen, just so that they’re aware of when they should expect the calls really. (Site K.3 Delivery staff)

Our findings indicate that successful delivery of remote home monitoring services depended on assessing how knowledgeable, equipped, and confident patients were to engage with a technology-enabled application or their telephone to provide readings. These factors had a varied impact on the nature of the relationships between delivery staff and patients. Delivery staff needed to be competent to rework risk knowledge using a nuanced understanding of a patient’s capability to share their lived experience through technologically enabled means. This reworking of risk, and subsequent actions by delivery staff, can be enhanced with technology that enables open exchange and mutual understanding between health professionals and patients.

## Discussion

This study found that staff involved in the delivery of COVID-19 remote monitoring services had to engage in interpreting a range of risk-based knowledge which influenced their ability to flexibly deliver both technology-enabled and analogue remote monitoring models. The process of reworking risk knowledge to reduce risk led to the creation of much ‘invisible work’, that is, translating monitoring data and ‘re-embedding’ the information into a useable format. Findings indicate that staff generally reported positive experiences of delivering the service and felt that it was easy to deliver and valued support. A range of training and support opportunities were provided but there was a perception that staff would have benefitted from further training to address the challenges associated with risk work given the different levels of risk for different clinical conditions. They would also have benefitted from support to improve the style and content of their consultations with patients to establish rapport and trustworthy social relationships, and establishing best practice on how to convey risk knowledge to colleagues to make clinical decisions.

### How findings relate to previous research

Given the rapid transition to using remote home monitoring models and technology to support people living with COVID-19 and long-term health conditions, our findings extend the literature on the training needs and experiences of staff asked to use such approaches within a wider context of workforce challenges across primary and secondary care.^20^ Staff understood that remote working differed from frontline, in-person care provision with patients but they were generally comfortable pivoting away from their usual mode of service delivery during the pandemic. However, the impact on how staff managed this transition varied, with some reporting an additional burden while others welcomed greater responsibility and the opportunity to support the wider health service response to the pandemic. For some delivery staff, remote home monitoring brought new challenges especially when conducting consultations over the telephone compared to face-to-face. In some cases, such a change led staff to reconsider their professional role, their identity, beliefs as well as capabilities when treating patients. Similar learning has been found in previous work,^21^ however, our study indicates that short-term staff-patient relationships established while providing care to treat COVID-19 was not jeopardised by remote monitoring.

Remote consultations are less embodied experiences for health professionals because they are unable to see and examine the physical body into conversation. For instance, remote home monitoring services can be interpreted as a ‘risk-based’ intervention where patient care is conceptualised through the lens of probabilistic accounts of the chances of an adverse acute event occurring. As part of a risk-based intervention, the nature of the work and the experience of delivery staff may be based on following precalculated risk thresholds. However, when such thresholds are absent or under constant review, staff may be uncomfortable making clinical risk-based decisions autonomously.^22^

Staff received a range of training and support to deliver remote monitoring models. Yet, a desire for bespoke training to reflect local organisational working meant that some staff may have been better equipped to work more independently than others. Frontline delivery staff were asked to rapidly transition from usual methods of providing face-to-face services to remote ways of working where the evidence of the effectiveness, benefits or challenges as well as staff experiences of remote home monitoring services was limited.^23^ Given the speed at which remote home monitoring services were implemented, this left a significant gap between staff skills, competences and confidence.

Staff demonstrated that they were capable of adapting, acquiring new skills and knowledge, and demonstrating a commitment to providing continuity of care, even where they were struggling with the increased responsibility attached to their adapted roles. Our study found workload differences for those balancing their workload with other commitments compared to those who were redeployed. This supports our earlier findings from the first wave of the pandemic, where staff were able to focus on delivery of remote home monitoring services due to cancellation of elective care or other activities, and the redeployment of staff.^12^ Delivery staff, particularly nurses, remained committed to taking a patient-centred approach to providing care that took account of the emotional and physical impacts of COVID-19 on patients. In addition, all staff delivering remote home monitoring models may have benefited from increased engagement with senior clinicians and managers in their respective organisations with regard to how models should be implemented locally.^24^

Given the range of skills required to deliver remote home monitoring models, an interprofessional, multidisciplinary approach is needed not only for addressing COVID-19 but for chronic disease management in general.^25^ A study of remote monitoring for COVID-19 patients in the USA showed that delegation of duties among teams involving senior physicians, nurses and administrative staff contributed to more effective implementation and higher levels of patient satisfaction, compared with a single practitioner-led model.^26,27^ In addition, staff require support on interpreting various forms of patient data collected remotely and how to best make clinical decisions within a context where there are pressures on time, staff shortages, and limited availability of supervision and oversight. The patient-facing nature of risk work, as part of remote monitoring models, requires delivery staff to have a unique set of interactive and communicative skills, which includes how to discuss the emotional and wider socio-cultural impact of illnesses, as well as the physical effects, remotely.

### Limitations and strengths

There were several strengths and limitations to our study. We used a mixed-methods approach to capture staff experiences delivering a novel innovation which was rapidly designed and implemented to treat patients with COVID-19 in England. Our findings are based on a large sample of participants from a range of disciplines working across primary and secondary care. However, variation between case study sites in terms of staff configuration, resources, and nature of remote monitoring models made it difficult to synthesise findings, and this may limit the extent to which transferable lessons can be drawn from this work. Further, the response rate to our survey was only 39% and it may be that staff less engaged in the delivery of the service were less likely to respond; our findings may thus not be representative of all staff views or experiences.

### Implications for policy, practice and research

Our findings highlight a number of lessons for policy and practice both on the treatment of COVID-19 patients specifically and for staff to consider when delivering care as part of remote home monitoring models for acute conditions where the risk of exacerbations is high. Current approaches to training may not be adequate to support clinical leads and delivery staff to undertake a wide range of ‘risk work’ when managing patients with COVID-19 and other acute conditions. Staff need the opportunity to be better informed for delivering the service; they should be able to access electronic resources as and when required, and receive bespoke training relevant to their service and organisation. Service leads should consider whether they need to introduce formal sign off mechanisms to ensure staff have been trained adequately and to a required standard. We found that delivery staff lacked confidence when communicating the risks associated with COVID-19 to patients. This means that organisations introducing remote home monitoring services should promote an interprofessional, multidisciplinary approach where delivery staff have ready access to clinical oversight, especially when a patient has deteriorated to a stage where escalation is required and to support triage.

There were several factors that influenced the delivery of COVID-19 remote monitoring services. Primary and secondary care organisations need to think carefully about staff capacity to deliver remote home monitoring services given redeployed staff will no longer be available when routine service delivery recommences. The COVID-19 pandemic has accelerated innovation across the health sector and changed understanding of the role of technology to monitor patients as part of acute care. Further guidance may be needed on how best to incorporate technology as part of a service and how to embed this as part of staff training.

Further research is needed to explore staff experiences of delivering care for COVID-19 and other health conditions using remote monitoring. In particular, there is need for work on the competency and skill set required to deliver remote monitoring services, how training should be designed, and how to best embed training during the set up and implementation of services across primary and secondary care.^23^

## Conclusions

Remote monitoring models can play an important role in managing a large number of patients with a range of acute and chronic health conditions. The successful delivery of remote service models depends on the right workplace infrastructure. Our study found high levels of acceptability for managing patients using remote models among staff but there remain challenges around confidence when making clinically-informed decisions and a dependency on clinical oversight. There is a need to invest in training to better equip staff delivering remote services.

## Supplemental Material

Supplemental Material - Staff experiences of training and delivery of remote home monitoring services for patients diagnosed with COVID-19 in England: A mixed-methods study Click here for additional data file. Supplemental Material for Staff experiences of training and delivery of remote home monitoring services for patients diagnosed with COVID-19 in England: A mixed-methods study by Manbinder Sidhu, Holly Walton, Nadia Crellin, Jo Ellins, Lauren Herlitz, Ian Litchfield, Efthalia Massou, Sonila Tomini, Cecilia Vindrola-Padros and Naomi Fulop in Journal of Health Services Research & Policy
