# Supplementary material for: Staff experiences of training and delivery of remote home monitoring services for patients diagnosed with COVID-19 in England: A mixed-methods study
Source: J Health Serv Res Policy. 2023 Jun 27;28(3):171–80. doi: 10.1177/13558196231172586 (PMC10300624; doi:10.1177/13558196231172586)
Supplement: Supplemental Material - Staff experiences of training and delivery of remote home monitoring services for patients diagnosed with COVID-19 in England: A mixed-methods study [file sj-pdf-1-hsr-10.1177_13558196231172586.pdf]

# Online Supplement

## S1. Methods and data collection instruments

## a) NATIONAL STAFF SURVEY

### Sample and recruitment

#### *Selection of sites*

Twenty-eight services were included in our national evaluation. Each site had a research lead (MS, CVP, HW, JB, IL, LH) to support data collection and act as an ongoing point of contact for the site.

To obtain maximum variation, we sampled services based on a range of criteria, including the setting (primary care or secondary care), type of model (pre-hospital, early discharge, both), mechanism for patient monitoring (paper-based, app, both), geographic location (across different areas of the country), timing of implementation (implemented since wave 1 of the pandemic or recently implemented) and involvement in the evaluation with the other evaluation partners (Imperial and IAU).

Sites were recruited through an expression of interest process whereby we presented our study at local and national meetings and asked sites to express interest in participating. Clinical Research Networks facilitated the setup of sites and local governance approvals. Some sites were identified through our phase 1 evaluation.

#### *Staff survey*

We conducted a survey of staff involved in delivering COVID-19 remote home monitoring services in the 28 services, including clinical leads, delivery staff and data staff.

Staff at participating sites distributed surveys to staff. All survey sites were asked to keep a record of the number of surveys they have sent out to determine staff response rates.

For the staff survey, staff received an email from their GP practice/hospital or other relevant networks (together with reminder emails) with a link to fill out an online survey.

### Measures

#### *Staff survey*

We developed staff surveys specifically for this study.

Different sets of questions were developed for different groups of staff (i.e., one survey for service leads and one survey for staff delivering the service) and steered by relevant literature.[16] The main purpose of the staff survey was to gather information on the staff involved in delivering COVID-19 remote home monitoring services, different set-up processes and models implemented, staff experiences of implementing these models, factors influencing delivery and staff perceptions of patient engagement with the service. As part of the survey, we also sought to explore different experiences of analogue vs tech-enabled models. The survey included several closed questions which focused on documenting staff experiences of setting up, managing, and delivering the service. These questions were followed by a single, open text question at the end to give staff the opportunity to share any wider thoughts. To reduce burden and maximise response rates, the online

survey was developed to take no longer than 15-20 minutes to complete. The survey was delivered using an online platform (REDCap).

Survey questions were reviewed, and sense-checked by our clinical advisory group, PPI group and our cohort of nurse and midwives recruited from National Institute for Health and Care Research (NIHR) 70@70 Senior Nurse and Midwife Leaders programme. The staff survey was piloted with a small number of sites (N=2). Piloting aimed to determine whether questions were appropriate and relevant, while identifying areas for further refinement prior to circulation nationally. In response to feedback, we amended some of the staff survey questions and response option wording to improve clarity, added response options, re-ordered questions and amended question format.

## Data collection

### *Staff survey*

Surveys were conducted with clinical leads, 'frontline' delivery staff and those involved in data collection and management ('data staff') with the aim to recruit as many staff as possible.

For the staff survey, staff were asked to use an online link, provided by site specific study co-ordinators, which led respondents to an information page providing background to the study, potential risks, and a description of data use to ensure informed and voluntary participation. It was emphasised that individual responses would be treated confidentially and reported anonymously. Staff were asked to tick a box to indicate their consent to take part in the study. Data collection took place between February and May 2021. Each site recorded the number of surveys distributed to staff to determine response rates.

## Data management

Staff surveys were returned to the research team electronically through REDCap (a secure web-based application) Data from the completed surveys were stored securely using password protected spreadsheets to which only members of the research team had access to.

## Analysis

Sites were characterised with respect to their population size, the proportion in urban versus rural areas, and the proportion in the most and least deprived areas (with respect to national quintiles). For sites based on Clinical Commissioning Group (CCG) areas we calculated these characteristics using publicly available data at lower super output area (LSOA) level mapped to CCGs, while for trust-based sites we used data derived from inpatient Hospital episode statistics (HES) admissions during the financial year 2019/20, in addition to web searches for the trust catchment populations.

The quantitative survey data were analysed using SPSS statistical software (version 25). Descriptive statistics, multivariate and univariate analyses were conducted to compare staff experiences of delivering the service across staff groups and service models, and patient experiences of the service across patient groups and service models (as reported by patients and carers). Open text responses relating to staff experiences of delivering the service were coded thematically and inductively. We offered to carry out site-specific analyses of patient experience data for participating sites.

# b) INTERVIEWS WITH STAFF

## Sample and recruitment

### *Site selection*

A smaller sample of the overall study sites were included as case studies in order to conduct a more in-depth analysis of staff experiences.

Seventeen of the twenty-eight sites were selected (based on expression of interest and purposively selecting according to our site selection criteria) as in-depth case study sites using the aforementioned criteria .

### *Staff interviews*

For staff interviews, we aimed to purposively sample one or two members of staff delivering the service, one staff member leading the service (operationally or clinically), and one staff member knowledgeable about service data collection/analysis at each of the 17 sites (note, one staff member may fulfil more than one role).

Participants for the staff interviews were approached through each case study site's contact person/gatekeeper. Potential interviewees were introduced to the researcher or asked to contact the researcher to take part. The researcher contacted these potential participants via email and sent them a participant information sheet. Participants were given 48 hours to review the information and ask questions about the study. If the participant agreed to take part in the study, they were asked to sign the consent form. An informed consent process using participant information sheets and written consent (scanned forms or typewritten/electronic signature) was used for recruitment to ensure informed and voluntary participation.

## Measures

### *Staff interviews*

Staff interview topic guides included questions for staff leading a service, staff delivering a service and staff involved in data. The staff topic guides included questions about their role, the origin of the model, the aims and goals of the model, resources and processes of the model, staff training, facilitators and barriers of implementation, patient engagement, adaptations, monitoring and evaluation, impact, and recommendations and sustainability.

## Data collection

The researcher arranged a time to carry out the interview. Each site had a different lead researcher who conducted the interviews and liaised with sites on an on-going basis. Interviews were conducted by six researchers (MS, CV, HW, LH, IL, JB). Interviews were carried out via telephone or an online platform (e.g., Zoom or MS Teams) as preferred by the participant. Data collection for interviews was conducted between February and June 2021.

## Data management

All interviews were semi-structured, audio recorded (subject to consent being given), transcribed verbatim by a professional transcription service (TP Transcription limited), anonymised and kept in compliance with the General Data Protection Regulation (GDPR) 2018 and Data Protection Act 2018.

## Analysis

For staff interviews, data collection and analysis was carried out in parallel and facilitated through the use of rapid assessment procedures (RAP) sheets as explained in Vindrola-Padros et al.[17] RAP sheets were developed per site to facilitate cross-case comparisons and per population (to make comparisons between sub-groups). The categories used in the RAP sheets were based on the questions included in the interview topic guide, maintaining flexibility to add categories as the study is ongoing.

***Interview topic guide:_Staff leads***

## Introduction

The interview should last around 45 mins, depending on how much you would like to say. We will ask questions about how your trust/CCG has implemented COVID care at home – by this we mean patient self-monitoring and recording of COVID symptoms either pre-hospital or after early discharge from hospital (also known in some places as COVID virtual wards). We will feedback the results of this evaluation to local and national NHS and public health services, and results will be made available to the general public too.

If you do not want to answer a question, you do not have to, and if you feel uncomfortable or tired we can stop the interview at any point. Have you got any questions before we start?

| ***Main question*** | ***Follow-up questions*** |
|---|---|
| **ROLE**<br>1. Can you tell me about your current role? | a. Length of time in your current role (both in the CO@H service and their usual role)<br>b. Key responsibilities – in relation to CO@H and in the 'usual' role |
| **ORIGIN OF THE MODEL**<br><br>2. Can you tell me the story of how COVID care at home started? | a. When did it start?<br>b. Who led its development?<br>c. Has the model been or applied to other conditions apart from Covid-19? If yes, which conditions?<br>d. When did it become fully operational?<br>e. How long did it take you to get the model up and running?<br>f. Which NHS colleagues/organisations in your area (primary care, secondary care, others) were involved with the setup and delivery of this model (NHS and non-NHS colleagues/organisations at regional and/or national level? |
| **AIMS AND GOALS OF THE MODEL**<br>2. What are the aims of the model and its main features? | a. Population served<br>b. Admission criteria<br>c. Characteristics of patient groups<br>d. Pre-hospital (referral from community), step down from ED or early discharge from hospital or some combination of these?<br>e. Where located – primary, secondary, community services, integrated care Trust<br>f. Is a digital platform being used for patients to enter their observations? Is the service being |

| | |
|---|---|
| | delivered 24 hours per day, 7 days per week?<br>g. Availability of other services at community or primary care level for Covid-19 relevant to testing, diagnosis, mental health |
| 3. What are the main goals/outcomes of the service/model?<br><br>*(Differentiate for each patient pathway, if they have more than one, at each site)* | a. Minimise patient mortality and morbidity<br>b. Early identification of cases of deterioration<br>c. Minimise attendance/reattendance to ED<br>d. Reduced length of stay<br>e. Other |
| **RESOURCES AND PROCESSES OF THE MODEL**<br>4. Can you talk me through the staff journey from a patient contacting their GP/ED/being discharged from hospital to being discharged from COVID care at home?<br><br>*(Differentiate for each patient pathway, if they have more than one, at each site)*<br><br>Only relevant for staff delivering COVID care on virtual wards:<br>Are patients provided oxygen and/or medications (e.g. dexamethasone and Low Molecular Weight Heparin (LMWH)) given to patients?<br><br>5. How were pulse oximeters purchased?<br><br>6. What is the current staffing arrangement used to deliver your model? | a. Application of admission criteria, and referral processes (variation by age, ethnicity, deprivation)<br>b. Patient triage<br>c. Distributing pulse oximeters to patient<br>d. Patient information and training<br>e. Patient monitoring (who is involved, what was monitored, and how)<br>f. Mechanisms used for patient data reporting (i.e. app, paper-based)<br>g. Tools for flagging deterioration<br>h. Escalation processes (including any criteria and thresholds, safety netting)<br>i. Patient discharge from ward<br>j. Signposting to wider services<br><br>a. What was the reasoning behind including oxygen and/or medication to patients (i.e. complexity of the patient's health condition, severity of symptoms)<br><br>a. Which pulse oximeters used (NHSEI or purchased their own)?<br>b. What proportion of pulse oximeters were returned and re-used?<br><br>a. *Number of staff/ pay band/grades* |

| | |
|---|---|
| | b. *Rota*<br>c. *Responsibilities*<br>d. *Any new additional staff been recruited e.g. working with volunteers to delivery oximeters*<br>e. *Redeployment of staff working elsewhere within the organisation*<br>f. *Training non-clinical staff to complete patient monitoring activity*<br>g. *Impact on staff morale/job satisfaction*<br>h. *Key changes which staff have noticed in their everyday working practices/current workload* |
| **FACILITATORS AND BARRIERS OF IMPLEMENTATION**.<br><br>5. Can you describe the experience of collaborating with other colleagues/orgs to set up and deliver the model?<br><br>6.. Did you draw on learning from any existing models of home monitoring?<br><br>7. How has the planning of a Covid-19 vaccination programme impacted upon the setting up/running of the CO@H model (if any)?<br><br>8. What factors have facilitated the implementation of the model during wave 2 of the pandemic?<br><br>9. What factors have been a barrier to the implementation of the model during wave 2 of the pandemic? | a. From within your organisation?<br>b. From other parts of the country? |
| **ADAPTATIONS**<br>10. [If model was developed between Mar and Aug 2020] Did you adapt the model after wave 1 of the pandemic? | If yes, how and why?<br><br>a. *Patient triage/risk stratification*<br>b. *Patient information and training*<br>c. *Patient monitoring*<br>d. *Mechanisms used for patient data reporting (i.e. app, paper-based)*<br>e. *Tools for flagging deterioration*<br>f. *Escalation processes*<br>g. *Patient discharge from ward* |

| | |
|---|---|
| | h. *Staffing model*<br><br>If no, why not? |
| **MONITORING AND EVALUATION**<br>12. What data are you collecting from patients at present to monitor service delivery?<br><br>13. Can you share your thoughts about the quality of the data being recorded?<br><br>14. Can you describe the nature of data you are collecting on patient safety concerns and Covid-19 related 'near misses'?<br><br>15. Have there been changes to the data you've collected since wave 1 e.g. protected characteristics from your patients at the point of onboarding?<br><br>16. What data or information, if any, would you have liked to have collected, but couldn't? | a. *Have these data been linked to other data sources?*<br>b. *Who is able to access these data?*<br>c. *How have these data helped you to monitor progress against your expected outcomes? How else have they used these data?*<br><br>Any concerns about missing data?<br><br>If yes, why were changes made?<br>a. *If no changes have been made, may you explain why?* |
| **IMPACT**<br>17. What impact, if any, has the introduction of the service/model had on delivery of other services within your own organisation?<br><br>18. What impact, if any, has the service/model had on tackling health inequalities and/or reaching high risk populations?<br><br>19. What impact, if any, has the service/model had on the wider health and care system? | a. Have there been any concerns about patient safety and/or near misses that have occurred since the service began? (If yes, prompt for an example)<br>b. Have there been any occasions of patients refusing treatment and/or dropping out? (If yes, prompt for an example and how it has been addressed) |
| **RECOMMENDATIONS AND** | a. *Sustainability of the models* |

| | |
|---|---|
| **SUSTAINABILITY**<br><br>20. What advice would you give colleagues, similar to yourself, attempting to implement similar models in other areas of the country?<br><br>21. What relationships have developed or been strengthened during the course of developing and implementing the service?<br><br>22. What resources (for examples, materials, staff training) have developed as a result of developing and implementing the service?<br><br>23. Are there any new institutional structures or policies that exist as a result of developing and implementing the service?<br><br>24. How transferable is the model to other conditions? | b. *Areas that need to be improved*<br>c. *Transferability of these models to other conditions?* |
| 25. Is there anything else important that you think we should know that I have not asked you? | |

***Interview topic guide_Staff delivery***

## Introduction

The interview should last around 45 mins, depending on how much you would like to say. We will ask questions about how your trust/CCG has implemented COVID care at home – by this we mean patient self-monitoring and recording of COVID symptoms either pre-hospital or after early discharge from hospital (also known in some places as COVID virtual wards). We will feedback the results of this evaluation to local and national NHS and public health services, and results will be made available to the general public too.

If you do not want to answer a question, you do not have to, and if you feel uncomfortable or tired we can stop the interview at any point. Have you got any questions before we start?

| ***Main question*** | ***Follow-up questions*** |
|---|---|
| **ROLE**<br>1. Can you tell me about your current role? | a. Length of time in current role (both in the CO@H service and their usual role)<br>b. Key responsibilities – in relation to CO@H and in the 'usual' role<br>c. Non-clinical staff- how have they |

| | |
|---|---|
| | been supporting the monitoring (any other part of the delivery) of CO@H?<br>d. Volunteers- what organisation are they working for? How did your involvement come about? How has your involvement changed (if at all) during the pandemic (or has it been curtailed)? |
| **AIMS AND GOALS OF THE MODEL**<br>2. What are the aims of the model and its main features? | a. Population served<br>b. Characteristics of patient groups<br>c. Pre-hospital (referral from community), step down from ED or early discharge from hospital or some combination of these?<br><br>Sub-set of questions for more senior delivery staff:<br><br>a. Where located – primary, secondary, community services, integrated care Trust<br><br>b. Availability of other services at community or primary care level for Covid-19 relevant to testing, diagnosis, mental health |
| **RESOURCES AND PROCESSES OF THE MODEL**<br>3. May you describe your experience of delivering the CO@H, specific to your role, from a patient contacting their GP/ED/being discharged from hospital to being discharged from COVID care at home?<br><br>*(Differentiate for each patient pathway, if they have more than one, at each site)* | a. Application of admission criteria, and referral processes (variation by age, ethnicity, deprivation)<br>b. Patient triage<br>c. Distributing pulse oximeters to patient<br>d. Patient information and training<br>e. Patient monitoring (who is involved, what was monitored, and how)<br>f. Mechanisms used for patient data reporting (i.e. app, paper-based)<br>g. Tools for flagging deterioration<br>h. Escalation processes (including any criteria and thresholds, safety netting)<br>i. Patient discharge from ward<br>j. Signposting to wider services<br><br>*Volunteer specific question:*<br><br>k. *How does your role in delivering CO@H fit more widely with regard to the* |

| | |
|---|---|
| 4.Can you share your thoughts about patient safety concerns and/or near misses that have occurred since the service began? Please provide examples of instances if possible. | *organisation running your volunteer programme (if outside the NHS)?*<br><br>*Only relevant for staff delivering COVID care on virtual wards and providing oxygen and/or medications e.g. dexamethasone and Low Molecular Weight Heparin (LMWH):*<br><br>I. *Can you describe the process administering oxygen and/or medications for patients?*<br>• *Which patients received oxygen and/or medication?*<br>• *How were patients provided oxygen and/or medication (e.g. at onboarding, drop off service)*<br>• *Was primary care informed?*<br>• *Describe any challenges you faced when administering oxygen and/or medications?*<br>• *How was the process of receiving oxygen and/or medications understood by patients (e.g. anxieties, reluctance)* |
| **STAFF TRAINING**<br>5.Describe how you felt when you learned about the use of the [name of remote monitoring platform) to treat patients and the approach your organisation's planned to use for its implementation?<br><br>6.Can you describe the training you have received to deliver the model to patients? What further training would you like? | a. Confidence about their own technological/digital literacy<br>b. Previous experience of using a new technology to treat patients<br>c. Have their attitudes changed towards the use of digital platfroms since using remote home monitoring?<br><br>a. *Familiarity with CO@H competency framework/any other framework or skills requirement- does this cover all needs?*<br>b. *Confidence about delivering the model to patients*<br>c. *Concerns about the nature of training received (or lack of)*<br>d. *Desire for greater oversight*<br>e. *Working through patient scenarios with members of the team* |

| | |
|---|---|
| 7.What skills, from your previous/existing role, have been useful when delivering this model of care to patients?<br><br>8.Describe the experience of working with new and/or existing members of staff in your organisation to deliver the model?<br><br>9.Can you describe the nature of support and guidance you received (if any) during the set up and delivery of the service from within your organisation? | a. *Challenges and tensions (e.g. working remotely, communication with new colleagues etc..)*<br>b. *Positives taken from delivering other services in the past*<br>c. *Level of comfort with (and satisfaction/enjoyment of) MDT working*<br><br>a. *Support from whom?*<br>b. *What type of support received?*<br>c. *Accessibility of support* |
| **PATIENT ENGAGEMENT**<br>10.Can you describe the nature of your engagement with patients referred to the service during wave 2?<br><br>11. What were your perceptions of using pulse oximetry remotely to manage patients with Covid-19? | a. Did you have trouble accessing any patient groups? Has there been any tailoring of the service to meet specific needs/requirements?<br>b. Do you feel patients and carers received all of the necessary information? Do you feel they understood the information?<br>c. Do you feel that you gathered enough information from your patient and carers in realtion to their wider social circumstances to understand how best to use remote monitoring for them?<br>d. How did patients engage with the service delivered throughout wave 2? Did some patients choose not to engage?<br>e. Did patients appear confident when describing how to use the oximeter/ record observations/ share concerns?<br>f. Did any patients appear anxious/need reassuring at any stage?<br>g. How would you describe your experience engaging with family members and/or carers of patients? |

| | |
|---|---|
| | a. *Level of confidence*<br>b. *Any tensions/ need for reassurance*<br>c. *Familiarity with digital platforms (or lack of); particularly remote consultations*<br>d. *Enhanced communication skills to undertake difficult conversations remotely* |
| 12.What advice would you give colleagues, similar to yourself, attempting to implement similar models in other areas of the country?<br><br>13.Is there anything else you think we should know that I have not asked you? | |

**STAFF SURVEY**
**Site ID: [xxx]**

| Group of questions | Questions to cover |
|---|---|
| **Is participant clinical lead/managers or staff delivering Co@H** | • Clinical lead/manager or staff delivering Covid home monitoring (please select) *(this will affect which questions are provided)* |
| Demographic characteristics- **ALL** | • Professional role and band / clinical, non-clinical, volunteer *for delivering staff only*<br>• What role have they played in Covid home monitoring *(i.e. set-up/management, referral, triage, patient monitoring, escalation and discharge).*<br>• How long have they been involved in Covid home monitoring<br>• When starting your role in covid home monitoring, did you have any relevant experience or were you in need of training from the beginning? *(select from these options in a drop-down. Plus 'had relevant experience but still required training.'*<br>• did they have any relevant experience ? or needed training from the beginning<br>• Whether they are working remotely or are in a hot hub?<br>• Whether they are sharing this role with other roles |
| Questions on the processes for covid home monitoring – **CLINICAL LEADS/MANAGERS ONLY** | • Where is the service managed?<br>○ Primary care<br>○ Secondary/hospital<br>○ Community services<br>○ Other<br>• Type of model<br>○ Pre-hospital<br>○ Step-down<br>○ Both<br>• Is the service being delivered 24 hours per day, 7 days per week? (Y/N)<br>• When was the service launched?<br>• Type of monitoring used *(Paper based/app/both)*<br>• How are patients identified and referred? *(select from: NHS 111, Covid Clinical Assessment Service (CCAS), Test and Trace, hot hub, GP, Emergency department, ambulance service)*<br>• What processes are involved in the model? *(select all that apply)*<br>○ Patient triage<br>○ Patient information and training<br>○ Patient monitoring<br>○ Tools for flagging deterioration<br>○ Escalation processes and referring to other services<br>○ Patient discharge from the ward<br>Questions about the model of care:<br>• Who is distributing pulse oximeters? *(clinical staff, non-clinical staff, volunteers, students, other)*<br>• Who is carrying out monitoring? *(Select all that apply: clinical staff, non-clinical staff, volunteers, students, shielding individual, other)* |

| | |
|---|---|
| | • How often do staff have contact with patients *(Select from: several times per day, once per day, weekly)*<br>• Which services are patients signposted to during or after using covid home monitoring? *(Select from: GP, community care, other)*<br><br>• What criteria are used to enrol patients to covid home monitoring? *(Select all that apply:*<br>o Diagnosed with COVID-19: either clinically or positive test result<br>o Symptomatic<br>o Aged 65 years or older<br>o Under 65 years and clinically vulnerable to COVID (e.g. late presentation, prior respiratory or chronic illness)<br>o Downs Syndrome<br>o Other Learning Disability<br>o BAME<br>o Obesity<br>o Other (please specify)<br>• |
| Staff experiences of delivering covid home – **DELIVERING STAFF ONLY** | • How have you found delivering Covid home monitoring? In particular: *(Likert scale – very easy to very difficult, N/A)*<br>o The triage processes<br>o Monitoring patients (e.g. using the app/paper-based system)<br>o Processes to escalate patients<br>o The IT systems you are using<br>o Working with other covid-related services |
| Training/support received – **DELIVERING STAFF ONLY** | • Do you feel adequately supported in your role? *(Yes/No)*<br>• Have you received training in your area of responsibility? *(Yes/No)*<br>• Are you confident in your ability to carry out your responsibilities *(Yes/No)*<br>• Questions about training needs:<br>o Have you completed the Covid home monitoring competency framework? *(Yes/ No)*<br>o Do you feel clear about your role, responsibility and accountability? *(Yes/No)*<br>o Do you feel that you have any further training or support needs? *(Yes, No)*<br>If yes, what do these relate to? *(select all that apply – clinical pathways, processes to triage, processes to monitor patients (e.g. documenting patient interactions), escalation, conflict resolution, use of IT systems, other)* |
| Training/support received – **CLINICAL LEADS/MANAGERS ONLY** | Questions about support:<br>• To what extent do you agree with the following statement: There is enough staff/capacity to deliver the service as intended? *(Likert scale from strongly disagree to strongly agree)*<br>• Have you received support from NHSEI (or others) regionally/nationally for the following? *(Select all that apply):*<br>o *Training for staff; training for patients; setting up/using systems for recording data; guidance for* |

| | |
|---|---|
| | *implementation; obtaining pulse oximeters; distributing pulse oximeters; other)*<br>• Are there any additional training resources/training required to deliver the service *(Yes/No)*<br>o If yes, what do these relate to? Clinical pathways<br>o Processes to triage<br>o Processes to monitor patients<br>o Processes to escalate patients<br>o The IT systems you are using |
| Impact of covid home monitoring on workload, and job satisfaction – **ALL** | • Questions on the impact of covid home monitoring on their work, including:<br>o Impact on staff workload – in addition or instead of their usual work?<br>o Impact on staff job satisfaction levels?<br>o Impact on stress |
| Staff perspectives on engagement and experiences of service users – **DELIVERING STAFF ONLY** | • How have service users engaged with/responded to the service? *(Likert scale – very poorly to very well)*<br>• How well do you think service users have engaged with/ used/ carried out the following *(very poorly to very well, N/A):*<br>o Taking measurements using pulse oximeters<br>o Providing readings over the phone<br>o Providing responses via app<br>• Do you think that service users have felt reassured by receiving CO@H? *(yes/no)*<br>• Are there any types or groups of service users facing barriers to accessing the service? *(yes/no)*<br>o If yes, which groups? *(select all that apply: BAME, patients with learning disabilities, elderly, non-English first language, cognitively impaired)*<br>o Has there been any tailoring of the service to accommodate specific needs/requirements? *(yes/ no)* |
| Impact - **ALL** | Do you think the service is having an impact on the following? *(select all that apply)*<br>o Reduce patient mortality<br>o Reduce patient morbidity<br>o Reduce health inequalities<br>o Increase health inequalities<br>o Early identification of cases of deterioration<br>o Reduce attendance/reattendance to ED<br>o Reduced use of ICU/ventilation<br>o Reduce hospital admissions<br>o Reduced length of stay in hospital<br>o Other |
| Data – **CLINICAL LEADS/ MANAGERS** | • What data are you collecting from patients at present to monitor service delivery? *(select all that apply)*<br>• Have these data helped you to monitor progress against your expected outcomes? *(Likert scale)* |
| Use in different settings – **CLINICAL LEADS/ MANAGERS** | • Have you used home monitoring for service users with other conditions as part of another service? *(yes/ no)*<br>o If yes, which conditions? *(select all that apply)* |
| Open text question - **ALL** | • Is there anything else you'd like to tell us about your experience of delivering/ managing Covid home monitoring? *(Please write in the box below)* |

# S2. Site characteristics

| Domain | Type of remote home monitoring | No. of sites (n=28) | No. of case study sites (n=17) | Response from survey (n, all sites/case study sites) | Response from interviews (n, case study sites only) |
|---|---|---|---|---|---|
| Type of model | CO@h (pre-hospital) | 13 | 9 | 135/109 | 30 |
| | Virtual ward (early discharge from hospital) | 4 | 1 | 33/12 | 4 |
| | Integrated CO@h and virtual ward | 11 | 7 | 124/78 | 24 |
| Setting | Primary care/community care | 16 | 11 | | |
| | Secondary care | 5 | 3 | | |
| | Both | 5 | 3 | | |
| | Not specified | 2 | 0 | | |
| Mode of patient monitoring | Analogue only | 7 | 3 | | |
| | Tech-enabled and analogue | 21 | 14 | | |
| Geographic location | South west England | 7 | 3 | | |
| | South east England | 5 | 4 | | |
| | East of England | 1 | 1 | | |
| | Greater London | 5 | 3 | | |
| | East midlands | 2 | 1 | | |
| | North east England | 2 | 1 | | |
| | North west England | 5 | 4 | | |
| | Yorkshire and Humber | 1 | 0 | | |
| Month service started | Before November 2020 | 11 | 7 | | |
| | In November 2020 | 13 | 9 | | |
| | After November 2020 | 4 | 1 | | |

# S3. Characteristics of staff survey respondents

| | Service managers/clinical leads (n=70) | Delivery staff (n=222) | Total (n=292) |
|---|---|---|---|
| Professional role within the service n (%) | | | |
| Clinical | 49 (70) | 157 (71) | 206 (71) |
| Non-clinical | 23 (33) | 63 (28) | 86 (29) |
| Student | 1 (1) | 4 (2) | 5 (2) |
| Other | 4 (6) | 0 | 4 (1) |
| Redeployed n (%) | | | |
| Yes | 7 (10) | 71 (32) | 78 (27) |
| No | 52 (74) | 131 (59) | 183 (63) |
| Not applicable | 11 (16) | 20 (9) | 31 (11) |
| Sharing COVID remote monitoring role with any other role(s) n (%) | | | |
| Yes | 52 (74) | 137 (62) | 189 (65) |
| No | 13 (19) | 78 (35) | 91 (31) |
| Not applicable | 5 (7) | 7 (3) | 12 (4) |
| Length of time involved in the service n (%) | | | |
| < 1 month | 1 (1) | 13 (6) | 14 (5) |
| 1-3 months | 14 (20) | 91 (41) | 105 (36) |
| 4-6 months | 37 (53) | 81 (37) | 118 (40) |
| 7-9 months | 13 (19) | 18 (8) | 31 (11) |
| > 10 months | 5 (7) | 19 (9) | 24 (8) |
| Where based for the service n (%) | | | |
| Remotely | 3 (43) | 106 (48) | 136 (47) |
| Hot hub | 8 (11) | 23 (10) | 31 (11) |
| GP practice | 6 (9) | 7 (3) | 13 (4) |
| Hospital | 15 (21) | 32 (14) | 47 (16) |
| Other | 11 (16) | 54 (24) | 65 (22) |
| Mode of monitoring n (%) | | | |
| Tech-enabled and analogue | 53 (76) | 173 (78) | 226 (77) |
| Analogue-only | 17 (24) | 49 (22) | 66 (23) |
| Aspects of the service involved with n (%) | | | |
| Development and/or piloting of the service | 50 (71) | 40 (18) | 90 (31) |
| Set-up and design | 53 (76) | 22 (10) | 75 (26) |
| Service management | 54 (77) | 32 (14) | 86 (30) |
| Referring patients | 30 (43) | 58 (26) | 88 (30) |
| Triaging patients | 29 (41) | 112 (51) | 141 (48) |
| Monitoring | 35 (50) | 199 (90) | 234 (80) |
| Escalation | 43 (61) | 162 (73) | 205 (70) |
| Discharge | 34 (49) | 156 (70) | 190 (65) |
| Monitoring/evaluating the service | 50 (71) | 61 (28) | 111 (38) |
| Other tasks (e.g. delivering equipment) | 3 (4) | 15 (7) | 18 (6) |

*Service leads n=70 and delivering staff n=222 unless specified

## S4. Characteristics of staff interview participants

| Staff characteristics | Interview participants (n=58) | |
|---|---|---|
| Gender | Female | 38 |
| | Male | 20 |
| Role in remote monitoring | Service lead | 23 |
| | Delivery staff | 28 |
| | Data lead | 7 |
| Job role categorisation* | Senior clinical lead/doctor equivalent roles | 18 |
| | Nursing and allied healthcare professional roles | 19 |
| | Administrative roles** | 4 |
| | Management/operational team roles | 16 |
| | Not known | 1 |
| Mode of monitoring used | Analogue only | 11 |
| | Tech-enabled | 47 |

Notes: *Job roles reported in interviews and categorised by lead authors (MS & HW), **e.g. administrators / healthcare assistants involved in monitoring

## S5. Results from the staff survey

Staff survey respondents reported a range of work-related tasks they had been involved with when delivering their respective services. The more frequently cited tasks included: monitoring activities (80%), escalation processes (70%), triage (48%), service evaluation (38%), development and piloting of the service (31%), service management (30%), and referral (30%) (Online Supplement S6). While almost half of service managers and delivery staff who responded to the survey felt that the service they provided had a positive or very positive impact (47%, n=127/266) on their workload, one-fifth reported that the opposite (21%, n=54/266).

Service manager survey respondents reported being supported in several ways by national government (NHS England)and local healthcare organisations and networks. For example: obtaining pulse oximeters (54%), receiving guidance for implementation of the service (43%), setting up and using systems for recording data (26%), support for training staff delivering the service (16%), distributing pulse oximeters (14%), and training for establishing patient engagement (7%). Most delivery staff (88%) reported that they felt they were adequately supported in their role within the service (Online Supplement S7).

In terms of training, 68% (n=200/292) of staff survey respondents reported having received training or training resources as part of their role. However, we found that the amount and nature of training received by staff was difficult to determine. Forty-one percent (n=28/69) of clinical leads/service managers and 12% (n=26/222) of delivery staff reported that they had further training or support needs beyond that already received (Online Supplement S7).

A key facilitator was determining the correct mix of staff that needed to be involved in the delivery of the service. Of the staff survey respondents, 71% (n=206/292) reported their role within the service to be clinical, 29% (n=86/292) non-clinical, and 2% (n=5/292) were students/volunteers (Online Supplement S7). In some sites only clinical staff were involved in monitoring patients, whereas others included a mix of both clinical and administrative staff. Volunteers were also used at some sites to support the administrative duties associated with setting up services.

Staff knowledge and confidence are important aspects when understanding the reworking of risk knowledge and risk minimisation by delivery staff. Most delivery staff survey respondents reported feeling confident or very confident in their ability to carry out their responsibilities (92%, n=204/222) and were clear about the requirements of their role (91%, n=202/221) (Table 4). Delivery staff reported few difficulties in completing tasks as part of delivering the service and most staff reported these tasks to be easy or very easy (range between 54% and 73% across tasks). Only a small number of staff cited difficultly completing certain such as using information technology and escalating patients (8% for each respectively) (Online Supplement S6).

Most delivery staff survey respondents reported high levels of patient engagement with their service (96%, n=209/219) and that service users had experienced few difficulties in using the oximeter (91% well/very well: n=193/212) and submitting readings over the phone (91% well/very well: n=186/205), with just over 20% experiencing some challenges submitting readings using an app (78% well/very well: n=134/172).

Some members of staff reported that their workload had increased more than initially expected (Online Supplement S7) as they were often dealing with wider determinants of ill health that were having a substantial impact on patient's well-being. For example, given the nature of the COVID-19,

many staff were supporting patients who had lost employment, were taking care of others diagnosed with COVID-19, or who also had mental health problems.

Overall, survey data indicated that most staff felt satisfied with the support and training that they had received; they also felt confident in delivering the service. Staff had an increased focus on understanding a patient's physical impairment. Hence, we found remote monitoring services can have the potential to be a reductionist service where the focus is treating COVID-19 with less attention on the wider context of patient's lives which may also be impacting their health and well-being. In addition, the introduction of remote monitoring platforms standardised what physical problems were relevant for delivery staff to identify.

# S6. Summary of staff experiences of delivering remote monitoring services

| **Findings from staff survey** | | **Responses** | **n (%)** |
|---|---|---|---|
| Support in delivering the service | Staff adequately supported in their role (n=221)* | Yes | 195 (88%) |
| | | No | 9 (4%) |
| | | Not sure | 17 (8%) |
| | Support provided by NHSE (or other organisations) (n=70)** | Obtaining pulse oximeters | 38 (54%) |
| | | Guidance for implementation | 30 (43%) |
| | | Setting up and using systems for recording data | 18 (26%) |
| | | Training for staff | 11 (16%) |
| | | Distributing pulse oximeters | 10 (14%) |
| | | Training for patients | 5 (7%) |
| | | Other | 2 (3%) |
| Staff reported experience of service | Confidence in carrying out daily responsibilities (n=222)* | Very confident or confident | 204 (92%) |
| | | Neutral | 15 (7%) |
| | | Not very confident | 3 (1%) |
| | Staff clear about the requirements of their role (n=221)* | Yes | 202 (91%) |
| | | No | 11 (5%) |
| | | Not sure | 8 (4%) |
| Staff reported experience of tasks* | Remote monitoring of patients (n=201) | Very easy or easy | 146 (73%) |
| | | Neutral | 47 (23%) |
| | | Difficult or very difficult | 8 (5%) |
| | Escalation processes (n=202) | Very easy or easy | 144 (71%) |
| | | Neutral | 42 (21%) |
| | | Difficult or very difficult | 16 (8%) |
| | Using IT systems (n=217) | Very easy or easy | 144 (66%) |
| | | Neutral | 56 (26%) |
| | | Difficult or very difficult | 17 (8%) |
| | Working with other COVID-related services (n=179) | Very easy or easy | 97 (54%) |
| | | Neutral | 73 (41%) |
| | | Difficult or very difficult | 9 (6%) |
| | Triage processes (n=181) | Very easy or easy | 114 (63%) |
| | | Neutral | 57 (32%) |
| | | Difficult or very difficult | 10 (6%) |
| Impact of service on workload, job satisfaction and work-related stress | Impact on workload (n=266) | Very positive or positive | 127 (47%) |
| | | Neutral | 85 (32%) |
| | | Negative or very negative | 54 (21%) |
| | Impact on job satisfaction (n=288) | Very positive or positive | 216 (75%) |
| | | Neutral | 63 (22%) |
| | | Negative or very negative | 9 (3%) |
| | Impact on work-related stress (n=267) | Very positive or positive | 60 (22%) |
| | | Neutral | 152 (57%) |
| | | Negative or very negative | 55 (20%) |

*Delivery staff only (i.e. responses do not include clinical leads or service managers)

**Service leads or managers only (i.e. responses do not include delivery staff)

# S7. Summary of additional training needs reported by staff

| **Additional training need** | **Service managers or clinical leads n (%)** | | **Delivery staff n (%)** | |
|---|---|---|---|---|
| | **Clinical role n=19** | **Non-clinical role n=12** | **Clinical role n=21** | **Non-clinical role n=5** |
| Clinical pathways | 17 (90%) | 11 (92%) | 15 (71%) | 3 (60%) |
| Processes for triage | 12 (63%) | 7 (58%) | 7 (33%) | 0 (0%) |
| Processes to monitor patients | 13 (68%) | 10 (83%) | 4 (19%) | 0 (0%) |
| Processes to escalate patients | 15 (79%) | 9 (75%) | 8 (38%) | 1 (20%) |
| The IT systems being used | 15 (79%) | 9 (75%) | 7 (33%) | 0 (0%) |
| Training on conflict resolution | - | - | 4 (19%) | 0 (0%) |
| Other | 2 (11%) | 0 (0%) | 2 (10%)<br>All mentioned ongoing support | 1 (20%) |

## S8. Training and support received by staff to deliver remote home monitoring services for COVID-19

- One off training sessions (virtual and face-to-face), delivered at the point where services were established, to delivery staff by senior clinical leads and service managers, as well as digital providers or case study site specific IT teams (e.g. virtual training sessions using dummy patients on applications)
- Receiving resources such as user guides and action cards for clinical and administrative staff
- Informal training on how to use digital applications as part of CO@H/CVW from a more experienced member of the service delivery team
- Scripts developed by individual service delivery teams with guidance on how to converse with patients and to familiarise the patient on how to engage with digital platforms
- Escalating questions to a service manager or more experienced staff either individually and/or during team meetings
- Development of a buddy system i.e. pairing experienced members of the team with less experienced/new members of the team
- WhatsApp group to share concerns with colleagues
- Daily briefing meetings to discuss patients on the service, changes in SOPs, and changes (if any) to national guidance of delivering virtual wards
